# Infectious diseases risk framing in Bulgarian media during early COVID-19 pandemic and the Ebola crisis

**DOI:** 10.1016/j.heliyon.2024.e36575

**Published:** 2024-08-20

**Authors:** Zhivka Getsova, Vanya Rangelova

**Affiliations:** a National Center of Infectious and Parasitic Diseases, Sofia, Bulgaria; b Medical University Plovdiv, Plovdiv, Bulgaria

**Keywords:** Content analysis, Media, Ebola, Covid-19, Disease outbreaks, Preparedness, Risk communication

## Abstract

**Introduction:**

Social forces, in conjunction with biological variables, play a crucial role in shaping the overall health of a community, particularly in the context of infectious disease outbreaks. Mass media calibrates risk perception among the public. The present study's aims are to review risk framings in the Bulgarian National Television in the early stages of the COVID-19 pandemic and to compare results with the communication strategies employed when Ebola was exported outside of Africa. The research seeks to provide a quantitative and qualitative understanding of how the media communicated risk during the two crises. It also aims to determine the extent to which messages altered based on the distinct epidemiological characteristics of the two epidemics.

**Methods:**

We used interdisciplinary analysis, combining methods from the social sciences and epidemiology. It is based on a controlled study of media content comparing the share and presentation of information on infections during two different outbreaks caused by newly emerging pathogens (in 2014 and 2020), as well as during periods with no specific concern for novel public health threats (JAN 2019 and OCT-NOV 2019). A content analysis was carried out.

**Results:**

The findings of the study indicate that during the Ebola crisis, medical frames were used in 92 % of the cases, whereas the majority of the analyzed media coverage of COVID-19 focused on the socio-political frame (97 %). During control periods, the extent of coverage using a medical framework varies between 100 and 86 %. In terms of geographic coverage, the presentation of content followed the principle of proximity. In non-emergency circumstances, clinical practitioners are often preferred candidates for interviews. However, during health crises, the media tends to highlight individuals holding administrative positions and authoritative functions.

**Conclusion:**

The present research confirms the hypothesis that public health emergencies increase the volume of infectious disease content on the news. The most frequently selected speaker categories should be briefed timely on outbreak developments in order to feed the media with accurate information.

## Hightlights

• Cumulative duration of news items in outbreak periods is nearly two-fold higher.
• 97 % of the COVID-19 coverage involved socio-political framings.
• Framings during the Ebola PHEIC referred predominantly to the medical focus.
• Pathogen novelty is a determining factor for the choice of speakers in the media.

## 1 Introduction

Together with biological factors, social forces may determine collective health, especially when it comes to outbreaks of infectious diseases. Since the swine flu (H1N1) pandemic in 2009, we have seen a few instances of public health emergencies of international concern (PHEIC) that developed in a very different context of digital information and rapid dissemination and interchange of news. Apart from the pathogens themselves, the ambivalent effects of heightened public concern have been an additional challenge for public health authorities to address.

Historically, lack of public awareness has always been regarded as a major danger to both collective and individual health. Therefore, communication strategies in public health have demonstrated their benefits during outbreak situations. Despite being considered to have a negative connotation, self-reported anxiety during epidemics has been viewed as an effective predictor of protective behaviours [1]. Studies on prevention practices during the early stages of COVID-19 also show that adequate risk perception motivates compliance with anti-epidemic measures [[2], [3], [4]]. Moreover, the public's concerns during the 2014 Ebola outbreak in Africa served as a catalyst for heightened interest in scientific research and political proactiveness [5]. Besides, evidence during the same Ebola outbreak suggests that media coverage may contribute to epidemic control by decreasing both the incidence peak value and peak time [6].

However, a negative spillover effect of excessive communication on epidemics (termed infodemic) has also been repeatedly reported. A study conducted during the H1N1 outbreak found that in periods of elevated psychological distress provoked by the increased awareness of the intensified spread of an infectious agent [1,7], emergency room (ER) visits tend to peak similarly as at times of disease prevalence [8], causing a burden on the health system before the critical phase of the outbreak. Furthermore, perceived exaggeration of the risk at a later stage may reduce sensitivity to health threats in the future [9].

Researchers studying the 2014–2016 Ebola epidemic in Africa have also established a relationship between the distorted perceptions of the disease – a result of both media coverage and informal information exchange, and the so-called fearonomic negative effects on the population's health [5,[10], [11], [12]]. Furthermore, evidence from the same PHEIC indicates that concentrating communication around a particular pathogen may reduce awareness of other infectious diseases. As a result of the public attention to the Ebola virus disease (EVD) crisis in Nigeria, which was one of the Ebola affected countries, the social sensitivity to other serious infectious diseases decreased [5].

A model using data from 11,926 disease outbreaks that occurred between May 2008 and 2009 has confirmed the significant impact of media in amplifying public concern. Depending on a disease's spread parameters and its perceived novelty and seriousness, researchers describe the media as a mighty factor capable of increasing levels of the peak mean social response by 40 %–100 % from baseline [13]. According to the model, higher values of spread parameters predict lower shares of media contribution in the social response observed, as social agents may come in contact with the infection through their social interactions and not necessarily through media coverage. This conclusion presents empirical data highlighting the nuanced distinction between the functions of traditional media and social media.

The power of media in the absence of an immediate real-life experience with a disease is illustrated by a survey among USA citizens whose knowledge of the Ebola epidemic was entirely shaped by the media [5,14]. Two-thirds of the respondents were concerned about a widespread epidemic in their own country, while more than 4 in 10 answered that they were worried about them or a family member falling ill.

The principle of pathogen novelty and assessed seriousness of disease that determines media saturation with content on infections was particularly valid during both the Ebola and the COVID-19 PHEICs. While there is an abundance of research on mental health in the context of the COVID-19 lockdown, there is a scarcity of publications that examine the role of media as a factor for public concern and mental health outcomes during health crises. A study describes a positive correlation between media usage for information on COVID-19 and anxiety symptoms [15]. Another study on the topic revealed that psychological distress in the US public followed the pattern of new infections until the end of the second wave when COVID-19 mortality went down [16].

Given the literature data and the ambivalence of the effect of increased public attention to infectious pathogens, more quantitative and qualitative research is needed on the content circulating through the media channels. Despite the widespread focus on social networks, it is the mass media that significantly shapes the discussion on social problems and calibrates risk perception among the public [[17], [18], [19]]. The present study aims to review risk framings in the Bulgarian National Television in the early stages of the COVID-19 pandemic and to compare results with the communication carried out when Ebola was exported outside of Africa. The research seeks to provide a quantitative and qualitative understanding of how the media carried out risk communication during the two crises. It measures the extent to which messages differed parametrically given the epidemiological specificity of the two outbreaks.

The research is important as it is the first piece of work that explores the role of media as a determinant of social perceptions during epidemics in the Bulgarian context. Such a topic has not been analyzed previously in the country. In addition, we have chosen to focus on content broadcasted by the Bulgarian National Television which is a public provider of information and is funded by the State. Therefore, the results may provide valuable information on how public media can serve better public health interests.

## 2 Methods

The paper suggests the approach of interdisciplinary analysis combining methods from the social sciences and epidemiology. It is based on a controlled study of media content comparing the share and presentation of information on infections during two different outbreaks caused by newly emerging pathogens (in 2014 and 2020), as well as during periods with no specific concern for novel public health threats.

Bulgarian public television central news emissions were screened for information on infections for 1 month in 2020 (January 2020) and 2 months in 2014 (October and November 2014). The selected screening periods correspond to the time of first reports on COVID-19 in China and globally and the moment the cases of the Ebola virus disease were registered outside the boundaries of the African continent where the initial outbreak took place. The month of October was characterized by the highest peak of Ebola-related content in the US prime-time [18]. These two periods in 2014 and 2020 are referred to as outbreak periods. The disparity in the two outbreak analysis period durations corresponds to the observed intensity of spread. Basic reproductive numbers are used to determine periods’ length for screening. The screening period for media content during the Ebola crisis is twice as long as the basic reproductive number of the pathogen tends to be up to two-fold lower (EVD 2014 R0 range (1.51–2.53), COVID-19 (1.5–3.5 from early 2020)) [20].

Similarly, the study analyzed news segments broadcasted in central news emissions in January, October, and November 2019, when no novel threat alerts were issued, referred to as control periods. Months in 2019 were selected to match months in 2014 and 2020 respectively (outbreak periods), as seasonality may influence the saturation of infectious disease media content.

To conduct a comprehensive analysis and comparison of news item shares, the time interval has been changed from minutes to seconds. This transformation allows for a more precise examination of the data, as it is now represented in a numerical format within an Excel spreadsheet rather than being expressed in a traditional time format. The calculations allowed percentages to be calculated.

### 2.1 A content analysis was carried out for all outbreak and control periods

Archived news broadcasts in video format were found available on the online website of the Bulgarian National Television (BNT) [21] and used for filling out a database with characteristics described below. Binary, continuous, and categorical variables were introduced to match the needs of the analysis.

Initially, using the model of a study conducted on prime-time Ebola narrative framing in the US media [18], it was decided to analyze the use of medical, socio-political, and human-interest frames used by the BNT central news emissions. The medical framework is defined as covering ten micro topics: symptoms, deaths, hospitalizations, mutations, vaccines, testing, preparedness, antibiotics, medical aid advice, and ventilation. The socio-political frame also consists of other ten micro topics: epidemic, masks, travel restrictions, other restrictions of social life, school closure, evacuation, hygiene, sick leave, sanctions, and local risk. The human-interest frame has been defined as involving first-person experience from a patient, citizen, or foreign citizen who has higher proximity to the problem. Each micro topic is considered a binary variable depending on whether it is covered by each of the news items reviewed. Following the initial pilot screening, it was agreed that a single news item might encompass multiple micro topics, given the complexity of journalistic narratives. Therefore, it is considered admissible for the different frames to appear within the same reportage as well, given the soft transition between subjects within the media settings. The frequency of micro topics is calculated. A verification procedure was conducted to assess if each news item encompasses at least one of the domains delineated under the medical or socio-political framework. Based on this premise, the frequency of each of the two frames is also assessed. The results are compared between the two designated outbreaks and control periods.

The number of news pieces involving selected categories of reputable information sources is compared for the two outbreak and control periods to understand the frequency with which each speaker category is interviewed by journalists for prime-time news emissions. The duration of reportages is taken down for each piece of news in the database, and the cumulative and average duration of news involving each category of speakers is calculated. Categories were predefined as follows: authority, clinical practitioner, expert, politician, foreign expert, and foreign authority. ANOVA has been performed to identify statistically significant differences in the durations analyzed.

The analysis also takes into account the main geographical focus of the information presented. It implements the indicator “local risk evaluation” for the infectious agents associated with certain novelty (Ebola and COVID-19) to categorically compare local risk framing. For that purpose, we count how many times risk is defined as either “minimal”, “intermediate”, or “real”. During the research, the database was also filled with information on other infectious diseases that made it to the prime-time news on the Bulgarian National Television. The positioning of items discussing infections in the leading summary of the central news emissions is also analyzed as a number for each selected period.

## 3 Coding

### 3.1 Six categories of reputable information sources have been identified for the purpose of this analysis

(One): “Authority” refers to a representative of a decision-making body with the legal power to impose social restrictions depending on the epidemic situation, such as the Ministry of Health or its subordinate Regional Health Inspectorates. (Two) “Clinical practitioner” refers to medical doctors who practice medicine and have direct contact with patients. (Three) “Expert” refers to representatives of national academic or scientific institutions who present evidence-based reports on epidemiologic dynamics. (Four) “Politician” refers to political figures other than the Ministry of Health who participate as sources of information representing political views. (Five) “Foreign Expert” refers to foreign representatives of academic or scientific institutions who present evidence-based reports on epidemiologic dynamics. (Six) “Foreign Authority” refers to foreign representatives of foreign decision-making bodies.

The coding process was carried out by a team of two independent coders. One of the authors acted as a coder and the contribution of the other one was intended as a control for the reliability of the coding performed. A control was conducted for the entire content under investigation and subject of this analysis. No measurement of intercoder agreement was introduced as a review of raw results and discussion between the coders preceded the creation of the database used for analysis. When disagreement occurred, each case was subjected to a discussion between the two coders and categorization was re-evaluated. Final categories were decided when the agreement was achieved.

## 4 Results

The total duration of news content related to infectious diseases in the two studied periods with outbreaks of interest (Ebola and COVID-19) is comparable. During January 2020, the total duration of reportages on infections was 55 min and 38 s, while during October and November 2014, it was calculated at 58 min and 23 s.

Similarly, the duration of infectious disease content is comparable between control periods. The study found that the cumulative duration of news items in control periods is lower: content referring to infections in January 2019 was 48 % of the content on infections in January 2020, and content in October and November 2019 was 47 % of the content in the same months in 2014. A detailed description of the duration per category can be found in Table 1.

**Table 1:** Duration of news content on infections during periods of screening.

Total duration of news (min. and sec.)	OCT-NOV 2014	JAN 2019	OCT-NOV 2019	JAN 2020
Pathogen of interest (% of total)	46:18 (79.3)	–	–	41:14 (74.1)
Other infections	12:05	26:39	28:34	14:24
… of them ARI/Flu (%)	04:48 (39.7)	22:15 (92.8)	06:55 (39.2)	11:11 (77.7)
Total (% of all infectious disease content screened 2014–2020)	58:23 (34.5)	26:39 (15.7)	28:34 (16.9)	55:38 (32.9)

In terms of geographical focus, in both the Ebola and COVID-19 study periods, the majority of content on all infections pertains to the imminent threats to the European continent (Table 2). However, when content on specific outbreak biological agents is analyzed geographically, during the Ebola outbreak most of it was focused on Europe (50.5 %) and a very small share commented solely on the situation on the continent of origin (1.5 %). Regarding the COVID-19 outbreak, a different pattern is observed. The majority of content discusses the situation on the continent of origin (39.7 %) (Table 3).

**Table 2:** Content on pathogens (Ebola and COVID-19) and content on Europe as duration, duration of European content on specific pathogen as duration and share of totals (pathogen and Europe).

	Pathogen total	Europe total	European content on pathogen	European content on pathogen as % of Pathogen total	European content on pathogen as % of Europe total
2014	46:18	35:28	23:23	50.5	65.9
2020	41:14	29:10	14:46	35.8	50.6

**Table 3:** Duration and share of outbreak pathogen content per continent of all content on outbreak pathogen.

	Europe (%)	Africa (%)	Asia (%)	World (%)
2014	23:23 (50.5)	00:43 (1.5)	–	22:12 (47.9)
2020	14:46 (35.8)	–	16:21 (39.7)	10:07 (24.5)

The data presented in Table 4 indicates that there is a significant decrease in focus on pathogens other than the two outbreak pathogens during outbreak periods.

**Table 4:** Duration per type of pathogen – outbreak or other pathogens.

Period	Outbreak pathogen	Other pathogens	Decline by
2014 (OCT-NOV)	0:46:18	0:12:05	136 %
2020 (JAN)	0:41:14	0:14:24	85 %
2019 (JAN)	–	0:26:39	–
2019 (OCT-NOV)	–	0:28:34 (0:20:03 in NOV)	–

In the 2019 control periods all content on infections is related to national settings, with the exception of a measles outbreak in Macedonia reported on January 4, 2019. In 2014, the geographical scope of news covering infections was wider, even when other infections different from the outbreak pathogen Ebola were presented. Examples include a case of nosocomial infections in the UK and the Influenza epidemic in the Netherlands.

The study's findings indicate that local risk was classified as minimal on three separate occasions throughout each outbreak monitoring period. The local risk from Ebola was reassessed as “real” one time, while the re-assessment for COVID-19 categorized the threat as an “intermediate” risk.

**Fig. 1:**
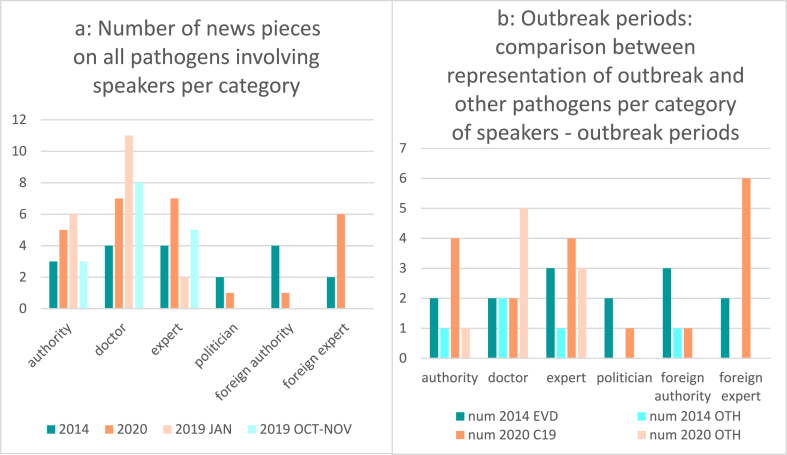
a: Frequency of news pieces on all pathogens broadcasted during the outbreak and control periods indicated involving speakers per category: authority, clinical practitioner (doctor), expert in infectious diseases, political figure, foreign authority, foreign expert.Fig. 1b: Outbreak periods: comparison between representation of outbreak and other pathogens per category of speakers during the indicated outbreak periods.

Analysis of the involvement of different categories of speakers shows that the same reportage may combine the shared views of representatives from different entities and backgrounds. Opinions have been observed to be complementary to each other rather than conflicting in all news items reviewed. Regarding all infections presented in 2014 (October and November), it was observed that speakers from the categories of clinical practitioners, experts in the field of infectious diseases, and foreign authorities prevailed. The same pattern with the inclusion of clinical practitioners and experts was observed in January 2020. In terms of the outbreak presentation, national experts and foreign authorities were the leading choices when it came to presenting the Ebola news. For COVID-19 foreign experts were the preferred source of information (Fig. 1а and 1b). During control periods, there is a higher prevalence of news articles featuring clinical practitioners, as opposed to epidemic periods, where speakers from political and international backgrounds are more frequently chosen. As anticipated, there was a lack of participation from foreign individuals or politicians during the control periods.

**Fig. 2:**
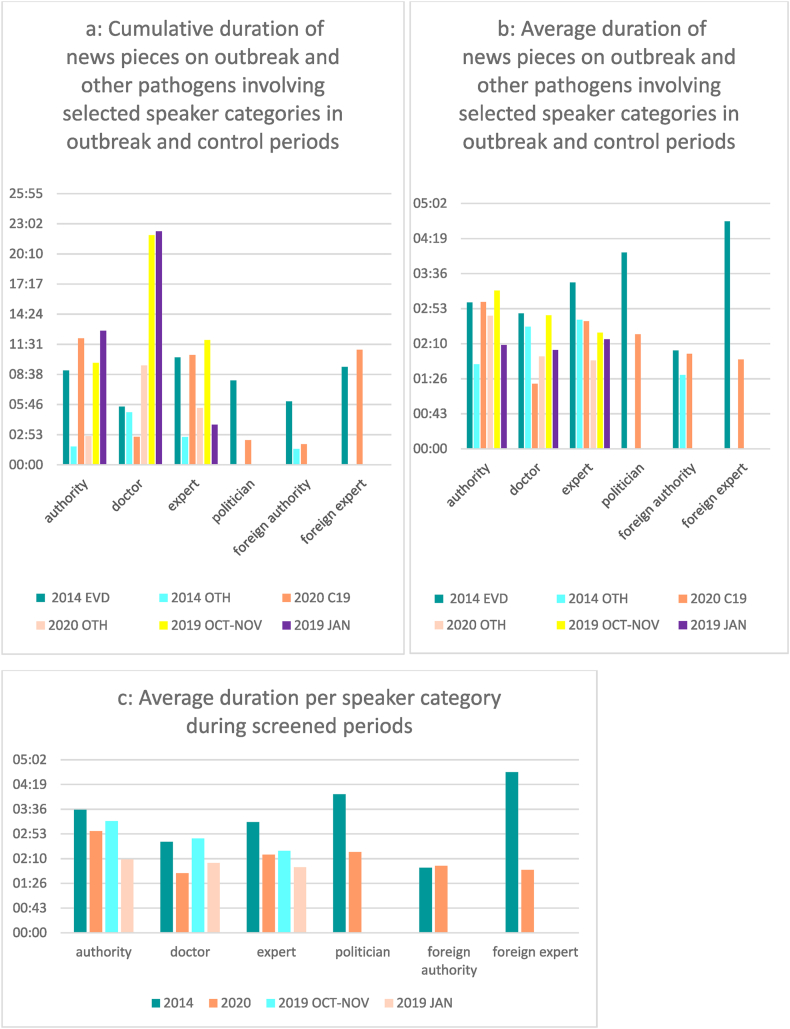
a: Cumulative duration of news pieces on outbreak and other pathogens involving selected speaker categories during indicated outbreak and control periods.Fig. 2b: Average duration of news pieces on outbreak and other pathogens involving selected speaker categories during indicated outbreak and control periods.Fig. 2c: Average duration of news pieces during screened periods per speaker category.

When the average duration of news items involving the different speaker categories is considered, it turns out that despite clinical staff being more present in news on other infections (different from the outbreak pathogen), less time is dedicated to news pieces involving clinicians. The average time dedicated to news pieces involving the different speaker categories is comparable to each other in control periods (Fig. 2a and b). The average duration is the highest for any of the speaker categories but the category of authorities when Ebola was in focus. Pieces involving foreign experts or politicians have a longer duration on average (Fig. 2b). During the first month of the spread of COVID-19, more time was dedicated to individual reports of the outbreak agent involving authority figures compared to all other speaker categories (Fig. 2b).

News pieces on outbreak pathogens involving national or foreign authorities in the two outbreak periods had a similar average duration. Reportage involving foreign experts and political figures had the longest average duration of news items in 2014 (Fig. 2b). The average duration of items involving authorities, clinicians, and experts was higher in October and November compared to January, as both results from the outbreak and control periods demonstrate (Fig. 2c).

A One-way ANOVA analysis of the reportage duration of involvement of the different categories of speakers found significant differences when the following categories were involved: authority and politician. Duration was observed to be higher when the two categories were involved compared to when they were not selected for speakers (Table 5).

**Table 5:** Mean duration of reportages involving and not involving selected categories.

	Mean duration when involved (N)	Mean duration when NOT involved (N)	P-value
Authority	02:50 (17)	01:54 (64)	0.004
Doctor	02:14 (30)	02:01 (51)	0.414
Expert	02:25 (17)	02:00 (64)	0.216
Politician	03:29 (3)	02:02 (78)	0.042
Foreign authority	01:54 (5)	02:07 (76)	0.724
Foreign expert	02:22 (8)	02:02 (73)	0.265

Analysis of data split by outbreak and control periods per speaker categories was also carried out. A One-way ANOVA found a statistically significant difference between durations depending on whether clinicians were involved or not during control periods (F (1,41) = 11.2 at p = 0.002). According to the evidence, the duration of news items in control periods tends to be higher when a clinician is interviewed in comparison to when not selected as a speaker. No significance was found for the difference observed in the average duration of reportages involving doctors during outbreak periods (F (1,36) = 0.132 at p = 0.718) but the opposite tendency was observed – reportages not involving clinicians being longer in duration.

Duration was calculated as higher for reportages involving the group of authorities in both outbreak and control periods (control periods: F (1,41) = 5.3 at p = 0.026 and outbreak periods: F(1,36) = 6.0 at p = 0.020). No significant difference regarding durations depending on the involvement of the speaker category was found for the other types of speakers analyzed.

Splitting data by time of the year and grouping news content from January and news content from October and November allowed for assessment of the seasonality effect in duration per speaker category. Results showed that content tended to be longer when authority figures were involved in January (F(1,43) = 9.3 at p = 0.004). No statistically significant results were obtained for the months of October and November or the other speaker categories.

**Fig. 3:**
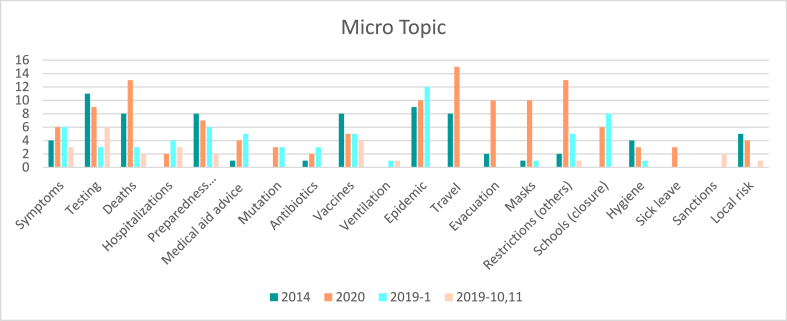
Frequency of micro-topics being addressed during the two PHEICs and two control periods.

The micro topic analysis found that 15 news items were very specific and covered only 1 of the selected micro topics. 17 items covered 2 of the selected micro topics and 49 covered 3 or more micro topics. Results on the frequency of micro-topic coverage show that restrictions, masks, and evacuation appeared 5 to 10 times more often in comparison with 2014 while testing, vaccines and preparedness were only 1.14 to 1.6 times more discussed in the screening period in 2014 compared to 2020. Travel was 1.88 times more discussed in the first month of the COVID-19 crisis compared to the Ebola emergency (Fig. 3). Concerns regarding mutations were only presented in 2020 in relation to the COVID-19 PHEIC. However, in the control period in January 2019, the same number of news items mentioned concerns about mutations with regard to influenza. Instructions for seeking medical advice were also more common in January 2020 in comparison with the 2014 outbreak period. However, during the Influenza season, such warnings of the need for medical attention are more common, as shown in the results from the control period in 2019. Coverage of vaccines, symptom awareness, testing and epidemic alerts is also common during control periods and appears most frequently referring to the influenza season.

Frame analysis shows that during the Ebola crisis medical frames were used in 92 % of the cases, while most of the analyzed media coverage of COVID-19 focused on the socio-political frame (97 %). During control periods, the coverage with a medical frame varies between 100 and 86 % (Table 6).

**Table 6:** Number of news items covering the selected frames and percentages of all content for the indicated period in brackets.

Frame	2014	2020	2019–1	2019–10,11
medical	23 (92 %)	22 (71 %)	12 (86 %)	11 (100 %)
socio-political	16 (64 %)	30 (97 %)	12 (86 %)	3 (27 %)
human interest	5 (20 %)	5 (16 %)	2 (14 %)	5 (46 %)

When framings are analyzed, it appears that most items cover simultaneously 2 frameworks, while few items cover all 3 analyzed frames at the same time (Table 7).

**Table 7:** Number of items that cover 1, 2 or 3 frames per the two outbreak and control periods studied.

N Frames	2014	2020	2019–1	2019–10,11
1	9	8	4	3
2	13	20	8	8
3	3	3	2	0

Regarding decisions for positioning of news items discussing infections within the news emission, in both October and November 2014 and October and November 2019 there were 4 instances of infections being included in the leading summary of news emissions. In January 2020 they were 5 vs. 3 in January 2019.

**Fig. 4:**
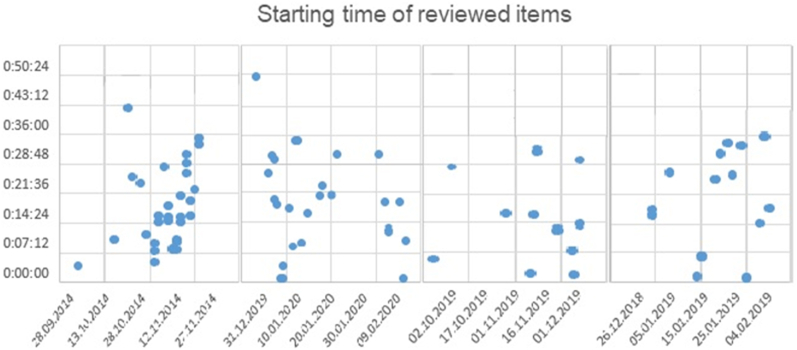
Starting time of news items within news emissions reviewed.

The earliest mean starting time of infectious diseases news was observed in the control period of October–November 2019–15:14 min (95 % CI: 07:54; 22:34). It is also the period in which the highest variance of starting time was observed. The outbreak period from January 2020 (COVID-19) was characterized by less variance in starting periods and ranked second for mean starting time: 18:04 min (95 % CI: 14:29; 21:39). During the Ebola outbreak in 2014, infectious diseases news tended to be reported a bit later on average – at minute 19:48 (95 % CI: 14:45; 24:50). The mean starting time in the control period January 2019 was at min 20:39 (95 % CI: 13:48; 27:30) (Fig. 4).

## 5 Discussion

The utilization of viral basic reproduction numbers enables the assessment of the spread parameters of the Ebola virus and SARS-CoV-2 based on existing literature data. This approach also facilitates the identification of observation periods that are approximately equivalent due to the objectives of this study [20]. However, given the complete novelty of COVID-19 in January 2020 and the certain familiarity of researchers with the Ebola virus’ gravity from previous outbreaks, the media is bound to address the two situations slightly differently. Prior to the analysis, it was decided that periods of screening should refer to a specific “turning point” of the outbreak, and therefore, the initial spread of the novel virus SARS-CoV-2 and the expansion of the EVD-affected regions were two comparable events in their meaning. In the two cases, the epidemic situation was considered sufficient criteria for media content screening.

The study of media content reveals that the duration of infectious disease content is similar between the two epidemic periods and the two control periods, despite the Ebola analysis period and its corresponding control period being twice as long. Evidence of cumulative outbreak periods’ duration being nearly twice higher in comparison to control periods suggests that pathogens associated with a certain degree of novelty raise public attention and therefore increase the share of infections in prime time news emissions. This finding confirms the observations of Reintsjes et al., Collier and Dan and Raupp [[22], [23], [24]]. Similar values of infectious disease content duration lengths in the Ebola and COVID-19 study periods of respectively two months and one month highlight the importance of spread parameters as a factor determining the degree of public attention [20].

Despite the expectation that control periods would not have the same total duration of news content due to their differing lengths (one month versus two months), the results obtained demonstrate that in January, BNT allocated a similar amount of time to discussing infections as it did in October and November combined. This can be attributed to the seasonal nature of the influenza virus, which typically peaks in the early weeks of the year. The duration in November is considerably higher than in October which aligns with the onset of the first rise of respiratory infections reflected in epidemic curves.

Other research found that information on influenza dominates the media in the months of October and January compared to November and December which in the case of Bulgaria has not been observed [25]. The difference between the volume of media coverage in October and November demonstrated when comparing our results with those from the US could be attributed to differences in the schedules of the national influenza vaccination campaigns. The trend of a sharp increase in infectious disease content that we described in January has also been observed in the research of Chen and Stoecker with regard to the flu epidemics. The teams of Saito and Olowokure also found a positive correlation between media saturation with content on influenza and the progress of the influenza epidemic [26,27].

The predominant geographical focus set by the Bulgarian National Television on Europe could be explained by the proximity principle [28] in the content selection practice. The same tendency is marked in a paper produced by Kim, who confirms the observation that the media often attempts to “internalize” crises caused by infectious outbreaks [29]. Pathogens associated with novelty in their spread cause the content of infections within the local European context to be dominated by alerts. The literature associates lack of novelty as a cause of decline of pathogen reporting by the media even during epidemic waves [22,30]. Interesting is the fact that of all Ebola content in 2014, more than half concerns the European geographical space, while in the case of COVID-19 in 2020 the European share is much lower (35.8 %). This finding can be associated with the specific risk framings during the initial phases of the two PHEICs. The results of the media monitoring exercise show that a higher degree of risk was attributed to the Ebola spread, defining the risk for Europe as “real”, while in January 2020 alerts from the Bulgarian National Television did not pass the threshold of “Intermediate”. Hooker, King and Leask describe similar reasoning for including the threat of resurgence of avian influenza in the Australian media in 2006–2007 based on geographic and cultural proximity to affected regions and elevated levels of risk of local spread [30].

The control periods primarily focused on national settings in terms of infections, except for a measles outbreak, which was also detected in a neighbouring country. Known pathogens with local circulation and epidemiology are discussed at a country and municipality level. However, in October and November 2014 (the Ebola outbreak period), on three occasions content on other infections different from the outbreak pathogen reporting epidemic information from abroad made it to the prime-time news in Bulgaria. This finding suggests that at times of intensified interest in an outbreak pathogen society may be more interested and sensitive to news concerning epidemic situations, which explains the choice of the media. However, more research is needed to confirm or reject this hypothesis as no such finding was found in the reviewed literature.

The selection of speakers in the media seems to be influenced by the level of novelty of the pathogen. The observed increase in the average duration of news items related to clinicians during control times, as compared to outbreak periods, can be attributed to their extensive clinical expertise in managing diseases caused by well-known microorganisms. Their involvement decreases during outbreak periods due to the novelty factor and lack of immediate hands-on experience within the national clinical practices. Expertise in these terms is rather sought from the appearance of foreign experts and authorities who share first-person observations on the threat and risk assessment findings. Resorting to foreign experience, however, could be seen as a challenge for communicating measures, according to qualitative research that took place in Switzerland during the COVID-19 pandemic. The public would always compare what is being done at a local level as public health measures which might be viewed as either too strict or too loose [31].

Politicians’ stances characterize outbreak periods and evidence shows that their involvement prevailed in 2014 compared to 2020. The journalistic choice of this category of speakers is most probably motivated by the need for a well-structured social response to the anticipated crisis. Expected instability in outbreak periods and higher risk perception in 2014 in comparison with 2020 matches proportionately increased political presence. Similarly, through authority involvement, the media exercises social control over the application of existing tools in the system for effective and efficient mitigation of the threat faced.

The control period in January when influenza seasonal circulation takes place is also characterized by the higher involvement of authority figures. The representatives of the Ministry of Health and its subordinate structures at the regional level (Regional Health Inspectorates) provide information on regulatory mechanisms that play a role in anti-epidemic control. As a known and regularly occurring epidemic, the influenza spread in January motivates the involvement of authority figures who are responsible for the management of the seasonal epidemic. Authority representatives' involvement peaks slightly during the Ebola and COVID-19 study periods due to social demands deriving from security concerns. Based on the literature research, the collaboration between the media and public health authorities has been recognized as advantageous in safeguarding public health. This collaboration promotes the development of trust, recognizes the importance of official communication channels, and improves the public's health literacy levels [22,31,32].

Analysis of the micro-topic coverage of the screened content shows that although the risk of COVID-19 local spread was framed as lower than the risk of Ebola, communication to the public stressed on anti-epidemic measures intended to stop transmission in the beginning. Restriction of social contacts and evacuation were tools intended to contain the epidemic and in line with the airborne mechanism of transmission. When the Ebola health crisis was presented, the focus was on long-term containment solutions such as vaccination and treatment. These observations may be explained by the longer history of the Ebola virus disease, which has brought about the need for the implementation of tools for infection control. Given the entire novelty of COVID-19 in January 2020 and the experience with SARS from 2002, short-term containment strategies ranked higher on the agenda [22]. Attention to vaccination, symptom awareness, testing, and epidemic alerts is registered at intermediate levels during control periods as well, which may be interpreted in terms of the needs of general health literacy [26].

Regarding framings, we find that the combination of different framings in the same news item is not an uncommon practice during outbreaks as the results of the analysis conducted during the SARS show [33]. The findings of the present work indicate that in January socio-political frames were more frequent which we interpret with the higher necessity of management of expected rise of acute respiratory infections such as influenza. While the COVID-19 PHEIC featured higher levels of sociopolitical content, framings during the Ebola PHEIC primarily referred to the medical focus. Such an outcome was also described during the SARS outbreak in China in 2003. Luther et al. explain the finding with the role of China on the economic market and the large investments associated with the business there [33]. Due to the origin of SARS-CoV-2 that caused the COVID-19 pandemic we believe that this is a prominent explanation for the difference we observed in Bulgarian news on Ebola and COVID-19.

The human-interest frame appears less frequently in comparison with the other two most used frames. It is also more common on average during the control periods compared to emergency situations when attention is understandably focused on medical and socio-political aspects that define organization and management of the crises. A review of the frames used by the media describes that the frames concerning health severity (in our case called medical frame), human interest and economic consequences (which we called socio-economic frame) are rarely explored jointly, which contradicts our observations [24]. Dan and Raupp observed that sub-themes pertaining to the socio-economic frame are a common characteristic for outbreaks caused by influenza but not so much by E. coli. Given the routes of transmission of the outbreak pathogens subject of the current analysis, it is expected and in line with previous international finding that the medical and the socio-economic frames prevail over the human-interest aspect [24]. The prevalence of the socio-economic frame in Bulgaria during the two outbreak periods characterized by high degree of novelty described in this paper matches conclusions drawn from the SARS outbreak where scientists saw an initial peak of such content with gradual decrease [34].

Interesting is a comparison with a similar study on US prime-time media coverage during the Ebola crisis [18]. Although the medical and socio-political frames appear to be more common in all broadcast sources analyzed from the US media like found in the Bulgarian National Television prime time, the share of coverage with the human-interest frame is similar only to the communications issued by CDC. Other broadcast programmes were found by US researchers to involve higher shares of human-interest content which can be explained through the infotainment approach used by American media.

The same study from the US also suggests that authoritative (“CDC-officials, federal, state or local officials”) and expert (“academic or medical experts”) voices dominated the representation of the Ebola outbreak. These findings are also consistent with the findings from Bulgaria.

Infectious disease news items in the BNT central news emissions that last on average around 40 min are presented most frequently in the middle of the programme broadcast. Evidence suggests that outbreak and control periods cannot be differentiated according to the positioning of infectious disease content.

## 6 Implications

Health emergencies increase infectious diseases content on the media. Therefore, media monitoring could be useful to obtain updates on the development of the epidemic situation. Given the sources used by journalists in the public television in Bulgaria, it is implied national media relies extensively on the national health system through its representatives, being the authorities, experts, and clinical healthcare staff, to cover infectious diseases topics. While in Bulgaria it is unlikely for the media to detect events happening at a national level before the responsible national structures, it may still provide information on regional outbreaks in their initial stages through interviews with clinical staff. In this regard, the media has the potential to get ahead of the formal institutionalized information flow. Although internal media monitoring is not expected to significantly contribute to national surveillance, it can be useful to foreign actors interested in taking stock of current progress in the country's epidemic situation.

Furthermore, media content screening may contribute with complementary information on signals at international level that might have been missed by infectious disease surveillance that is mainly focused on the country. In this aspect it is expected to be a reliable information source to surveillance activities as it uses foreign reputable and experienced sources to present infectious diseases events.

The results from the analysis on media framings carried out also support the hypothesis that media monitoring adds value to surveillance. The study suggests specific micro topics might be informative to surveillance at a different extent during outbreak and non-outbreak periods. Analysis of starting times suggest that screening should cover the emissions in their entirety.

A) The medical framework was found to be present in both outbreak and control periods which implies journalistic narratives may offer complementary epidemiological findings and enrich public health awareness. The elements through which they are predominantly presented (testing, mutations, mortality, and vaccination) suggest the content may contribute to understanding the epidemiologic context defined by the causative agent and level of disease spread and severity. Reporting on vaccinations may provide insights on population susceptibility during outbreaks caused by vaccine preventable pathogens.
B) The other two frameworks – the socio-political and human-interest ones, that were found present in the news content unfold opportunities for activities related to social listening. The observed reporting on elements such as travel restrictions, social restrictions and preventive behaviours (mask usage) implies media narratives are also an important source of behavioural and social change information.

## 7 Conclusion

The present research confirms the hypothesis that public health emergencies increase the volume of infectious disease content on the news. The framings adopted by the media influence the public's perceptions about the level of risk and the threat's nature. This work found evidence that together with the medical framework, the media develops infectious disease content through a socio-political and human-interest lens but does not implement the infotainment strategy like their US counterparts.

The analysis of the speakers selected by the journalists proves that clinical practitioners are a preferred choice for interviews outside of emergencies while during health crises the media gives priority to individuals with management functions and authoritative positions.

The present study demonstrates that the media employs the principles of pluralism as a variety of speakers from different backgrounds support the information on infectious diseases that is communicated to the public through the small screen. Journalists adjust their speaker list according to the known evidence of the pathogen of concern and respond adequately to the novelty challenge. The analyzed content shows that the Bulgarian National Television is aware of the role the media has in educating people on public health subjects. The micro topics touched on during the reportages approach the problems from an expert perspective but at the same time deliver accessible information on the most relevant to individual and public health aspects.

Findings on the discourse used and the speakers’ selection made by the media are important for risk communication effectiveness. Scientific knowledge on this subject allows for future improvements in aligning content presented to the public with official positions to avoid possible inconsistencies in presentations that erode social trust and thus, discredit public health interventions.

The research provides explicit information that identifies the most prevalent groups of individuals interviewed in the media. These figures should be considered for inclusion in internal institutional information flows and briefed timely on outbreak developments to feed the media with accurate information. The findings on frameworks used by the media can also be used for the development of guidelines for public speaking on infectious diseases.

## Limitations

The study has several limitations. Only content from the Bulgarian National Television was screened and analyzed. The source was selected as a public channel of information that receives public funding. No data was analyzed for private channels.

Although duration of news pieces has been indicated for each news item in the database, a variety of voices from different expertise commenting on one and the same event often mix within the same reportage and do not allow researchers to calculate exact time of TV appearance per category. That is why this analysis discusses only frequencies and duration of items involving the selected categories.

## Data availability statement

Has data associated with your study been deposited into a publicly available repository? – Yes. The research uses publicly available data from online broadcast archives.

## CRediT authorship contribution statement

**Zhivka Getsova:** Writing – original draft, Methodology, Investigation, Conceptualization. **Vanya Rangelova:** Writing – review & editing, Validation, Funding acquisition.

## Declaration of competing interest

The authors declare the following financial interests/personal relationships which may be considered as potential competing interests:Zhivka Getsova reports financial support was provided by Operational Program Science and Education for Smart Growth 2014–2020. If there are other authors, they declare that they have no known competing financial interests or personal relationships that could have appeared to influence the work reported in this paper.
